# *CEACAM* Gene Family Mutations Associated With Inherited Breast Cancer Risk – A Comparative Oncology Approach to Discovery

**DOI:** 10.3389/fgene.2021.702889

**Published:** 2021-08-10

**Authors:** Anna L. W. Huskey, Isaac McNeely, Nancy D. Merner

**Affiliations:** ^1^Department of Pathobiology, College of Veterinary Medicine, Auburn University, Auburn, AL, United States; ^2^Department of Drug Discovery and Development, Harrison School of Pharmacy, Auburn University, Auburn, AL, United States

**Keywords:** breast cancer, canine mammary tumor, *CEACAM*, whole genome sequencing, comparative oncology, inherited risk, rare protein truncating variants, splice mutations

## Abstract

**Introduction:**

Recent studies comparing canine mammary tumors (CMTs) and human breast cancers have revealed remarkable tumor similarities, identifying shared expression profiles and acquired mutations. CMTs can also provide a model of inherited breast cancer susceptibility in humans; thus, we investigated breed-specific whole genome sequencing (WGS) data in search for novel CMT risk factors that could subsequently explain inherited breast cancer risk in humans.

**Methods:**

WGS was carried out on five CMT-affected Gold Retrievers from a large pedigree of 18 CMT-affected dogs. Protein truncating variants (PTVs) detected in all five samples (within human orthlogs) were validated and then genotyped in the 13 remaining CMT-affected Golden Retrievers. Allele frequencies were compared to canine controls. Subsequently, human blood-derived exomes from The Cancer Genome Atlas breast cancer cases were analyzed and allele frequencies were compared to Exome Variant Server ethnic-matched controls.

**Results:**

*Carcinoembryonic Antigen-related Cell Adhesion Molecule 24* (*CEACAM24*) c.247dupG;p.(Val83Glyfs^∗^48) was the only validated variant and had a frequency of 66.7% amongst the 18 Golden Retrievers with CMT. This was significant compared to the European Variation Archive (*p-*value 1.52 × 10^–8^) and non-Golden Retriever American Kennel Club breeds (*p-*value 2.48 × 10^–5^). With no direct ortholog of *CEACAM24* in humans but high homology to all CEACAM gene family proteins, all human *CEACAM* genes were investigated for PTVs. A total of six and sixteen rare PTVs were identified in African and European American breast cancer cases, respectively. Single variant assessment revealed five PTVs associated with breast cancer risk. Gene-based aggregation analyses revealed that rare PTVs in *CEACAM6*, *CEACAM7*, and *CEACAM8* are associated with European American breast cancer risk, and rare PTVs in *CEACAM7* are associated with breast cancer risk in African Americans. Ultimately, rare PTVs in the entire *CEACAM* gene family are associated with breast cancer risk in both European and African Americans with respective *p-*values of 1.75 × 10^–13^ and 1.87 × 10^–04^.

**Conclusion:**

This study reports the first association of inherited *CEACAM* mutations and breast cancer risk, and potentially implicates the whole gene family in genetic risk. Precisely how these mutations contribute to breast cancer needs to be determined; especially considering our current knowledge on the role that the *CEACAM* gene family plays in tumor development, progression, and metastasis.

## Introduction

Breast cancer is a serious health concern. Amongst both sexes, it globally ranks as the second most commonly diagnosed type of cancer and the second leading cause of cancer-related deaths, accounting for ∼2.1 million new diagnoses and 626,679 deaths in 2018 (Bray et al., 2018). Worldwide, it is also the most common cancer diagnosed in women and the overall leading cause of cancer-related female deaths (Bray et al., 2018). In the United States, 2020 estimates predicted breast cancer to be the leading site of new cancer diagnoses in women and the second leading cause of cancer-related deaths, resulting in 276,480 new diagnoses and 42,170 deaths (American Cancer Society, 2020). Advances in breast cancer research have translated to better disease screening, diagnosis, and treatment, but new research questions continuously arise as time and medical needs progress (Cardoso et al., 2017).

Comparative oncology, which is the study of cancer biology and therapy in spontaneous, naturally-occurring cancers in companion animals, provides valuable models of human cancer that have and will continue to make research advances (Garden et al., 2018). Recent studies comparing canine mammary tumors (CMTs) and human breast cancers have revealed notable tumor similarities, identifying shared expression profiles and acquired mutations (Liu et al., 2014; Ettlin et al., 2017; Lee et al., 2018, 2019; Kim et al., 2019; Gray et al., 2020). CMTs can also provide a model of hereditary breast cancer susceptibility in humans, especially considering similar genetics and familial clustering (Goebel and Merner, 2017; Gray et al., 2020). While most CMT studies investigating inherited risk have focused on identifying genetic variants in orthologs of known human breast cancer risk genes (Goebel and Merner, 2017; Huskey et al., 2020), in this study, we investigate breed-specific whole genome sequencing (WGS) data in search for novel CMT risk factors. WGS studies have been used to make numerous disease gene discoveries in dogs, many of which clearly translated to human health (Gilliam et al., 2014; Guo et al., 2014; Sayyab et al., 2016; Kolicheski et al., 2017; Fyfe et al., 2018; Meurs et al., 2019). Taking a similar approach, we identified a *Carcinoembryonic Antigen-related Cell Adhesion Molecule 24* (*CEACAM24)* protein-truncating variant (PTV) in a Golden Retriever CMT pedigree, which ultimately revealed that rare PTVs in the *CEACAM* gene family are associated with breast cancer risk in humans. Aberrant expression of many *CEACAM* genes have previously been associated with tumorigenesis, and *CEACAM* gene products are recognized as clinically-relevant tumor markers (Kuespert et al., 2006; Beauchemin and Arabzadeh, 2013; Han et al., 2020). This is the first association to be reported between *CEACAM* gene mutations and inherited cancer risk.

## Materials and Methods

### Golden Retriever Pedigree and WGS

As previously described by Huskey et al. (2020), blood- or buccal-derived DNA samples were obtained from 18 CMT-affected Golden Retrievers from the Canine Health Information Center (CHIC) DNA repository, and a pedigree was constructed linking all 18 dogs in one large pedigree. Five of those Golden Retrievers (three females and two males) were selected for WGS. This number was influenced by the cost of WGS. Furthermore, aiming to identify breed-specific mutations, distantly related dogs were selected, including two males since male breast cancer is associated with hereditary disease (Huskey et al., 2020). The WGS data was processed through a bioinformatics pipeline (Huskey et al., 2020). Upon alignment to the CanFam3.1 reference genome and annotation using gene predictions from Ensembl build version 75, a script was written to isolate PTVs found in all five Golden Retriever samples. PTVs were defined as single nucleotide variants (SNVs) that resulted in a premature stop codon or abrogated a splice site, and small insertions or deletions (indels) that changed a transcript’s reading frame. Upon filtering, the genes with PTVs were classified into two different groups, orthologs of human genes or non-orthologs. Polymerase chain reaction (PCR) and Sanger sequencing were carried out to validate the PTVs in human orthologs. *CEACAM24* c.247dupG;p.(Val83Glyfs^∗^48) was the only validated variant. Following validation, the 13 remaining CMT-affected Golden Retrievers underwent PCR and Sanger sequencing to determine their mutation status.

### Canine Controls

As a convenient, publically available, online canine genetic variant repository, the European Variation Archive^1^ was initially used to note the allele frequency of *CEACAM24* c.247dupG;p.(Val83Glyfs^∗^48). The European Variation Archive provides high quality WGS variant calls of over 200 dogs from multiple breeds (breed and sex information was unknown). The data was obtained through Ensembl by accessing the canine gene’s “Variant table” under “Genetic Variation”; for a particular variant, “Population genetics” information was given, including European Variation Archive allele frequencies (Zerbino et al., 2018). Furthermore, additional splicing, frame-shifting, and stop gain mutations within the other dog *CEACAM* genes were investigated through Ensembl transcripts (*CEACAM16*: ENSCAFT00000044174; *CEACAM18*: ENSCAFT00000004587; *CEACAM20*: ENSCAFT0 0000047731; *CEACAM24*: ENSCAFT00000047960; *CEACAM28*: ENSCAFT00000022623). *CEACAM1*, *CEACAM23*, and *CEACAM30* did not have variant information available in Ensembl for European Variation Archive data.

Through the CHIC repository, blood or buccal-swab derived DNA from purebred, American Kennel Club registered dogs were randomly selected and obtained to determine the frequency of *CEACAM24* c.247dupG;p.(Val83Glyfs^∗^48). This included DNA from Golden Retrievers (*n* = 87), as well as 13 other breeds, including Petit Basset des Griffon (*n* = 10), Gordon Setter (*n* = 8), Australian Cattle Dog (*n* = 10), Siberian Husky (*n* = 10), Dalmatian (*n* = 10), Irish Setter (*n* = 9), Welsh Pembroke Corgi (*n* = 10), Standard Schnauzer (*n* = 10), Newfoundland (*n* = 10), Keeshond (*n* = 10), Great Dane (*n* = 8), Doberman Pinscher (*n* = 10), and Boxer (*n* = 10). PCR and Sanger sequencing were carried out to determine *CEACAM24* c.247dupG;p.(Val83Glyfs^∗^48) genotypes of each dog.

### Canine Statistical Analyses

Upon determining *CEACAM24* c.247dupG;p.(Val83Glyfs^∗^48) allele frequencies, *p*-values were generated using the Fisher’s Exact Test in R (v 3.5.1), comparing allele differences in Golden Retriever to control dogs, including both European Variation Archive and CHIC DNA samples.

### Dog and Human CEACAM Protein Analyses

EMBOSS water alignment (Madeira et al., 2019) was carried out to determine the level of homogeneity between the dog CEACAM24 protein and other dog and human CEACAM proteins. Additionally, InterPro (Hunter et al., 2009) and the Eukaryotic Linear Motif (ELM) resource (Kumar et al., 2020) were used to identify CEACAM domains and binding motifs, respectively.

### Human *CEACAM* Gene Analysis – The Cancer Genome Atlas

Due to the homogeneity of the CEACAM gene family and no direct ortholog of dog *CEACAM24* in humans, all human *CEACAM* family genes were investigated for rare PTVs in The Cancer Genome Atlas (TCGA) breast cancer cohort. Investigating inherited risk, only blood-derived exomes of breast cancer cases were analyzed. Overall, whole-exome binary sequence alignment mapping (BAM) files were downloaded using the Genomic Data Commons (GDC) Data Portal Repository through approved research project #10805. To acquire the samples, the specific filters under the “Cases” category included: Project (TCGA-BRCA), Samples Sample Type (Blood Derived Normal), and Race (“Black or African American” and “White”). The samples were further filtered under the “Files” category, including Experimental Strategy (WXS) and Data Format (BAM). A total of 170 sample files were obtained for African Americans and 650 for European Americans. These files were downloaded using the GDC Data Transfer Tool (version 1.2.0). Only individuals with known ages of breast cancer onset were used in this study; as a result, one European American and two African American BAM files were removed from further bioinformatics processing and statistical analysis.

The downloaded BAM files, which had previously been aligned to the hg38 human reference genome, were processed using the remaining steps of a pipeline adapted from the Genome Analysis Toolkit’s (GATK’s) best practices pipeline (Van der Auwera et al., 2013). Base quality scores were recalibrated using BaseRecalibrator and then HaplotypeCaller was used to generate genome variant calling format (gVCF) files (GATK version 3.6). GenotypeGVCFs was used to merge the individual gVCF files based on ethnicity (GATK version 3.6). The European American files were merged in batches of approximately 200 using GATK’s (version 3.6) CombineGVCFs prior to merging into a single VCF file with GenotypeGVCFs. The two ethnic specific VCF files were then processed through a variant quality score recalibration using VariantRecalibrator (GATK version 3.6), and, as recommended, SNVs were filtered using a pass filter of 99.5%, and indels were filtered using a slightly lower pass filter of 99.0% (Van der Auwera et al., 2013). Variants in *CEACAM1* (NM_001184815; chr19:42507306-42528481), *CEACAM3* (NM_001815 at chr19:41796587-41811554), *CEACAM4* (NM_001817; chr19:41618971-41627074), *CEACAM5* (NM_004363; chr19:41708626-41730421), *CEACAM6* (NM_00 2483; chr19:41755530-41772210), *CEACAM7* (NM_006890; chr19:41673303-41688270), *CEACAM8* (NM_001816 at chr19: 42580243-42594924), *CEACAM16* (NM_001039213; chr19:4469 9151-44710718), *CEACAM18* (NM_001278392; chr19:5147 8643-51490605), *CEACAM19* (NM_020219; chr19:44671 452-44684355), *CEACAM20* (NM_001102597; chr19:44506159-44529675), and *CEACAM21* (NM_001098506; chr19:41576 166-41586844) were then extracted from the ethnic specific VCF files and annotated using ANNOVAR (version June2017). Variants were filtered to include rare PTVs with ethnic-specific minor allele frequencies of <1% in Exome Variant Server (EVS; National Heart, Lung, and Blood Institute (NHLBI) Exome Sequencing Project) (Exome Variant Server, 2019).

### Human Statistical Analyses

Using the Fisher’s exact test (Sprent, 2011) in R (v 3.5.1), individual PTVs were assessed to compare allele frequency differences between ethnic-specific TCGA breast cancer cases and EVS controls. The Fisher’s method was used for gene-based and gene family-based aggregation analyses (Fisher, 1925; Sutton et al., 2000). The R tool “sumlog” (in the “metap” package) was used to combine *p-*values for each aggregation test. To accommodate for the one-sided nature of the Fisher exact test *p*-values, compliments of *p-*values in the opposite direction were used in the calculations for the Fisher’s method aggregation analyses.

### Human Mutation Analysis

Mutalyzer was used to determine the effect of frame-shifting and non-sense variants on the coded protein (Wildeman et al., 2008). Human splicing mutations that affected non-protein-coding exons of the mRNA, specifically in the 3′ untranslated region (UTR), were analyzed using the miRDB tool to identify microRNA binding sites potentially lost due to a splicing mutation (Chen and Wang, 2020). For each gene harboring a splice mutation affecting non-protein-coding exons, microRNA binding sites within the 3′ UTR with a target score of ≥80 were noted. The top five ranked microRNA targets were investigated for previous cancer (specifically, hereditary breast and ovarian cancer (HBOC) syndrome) associations.

## Results

**FIGURE 1:**
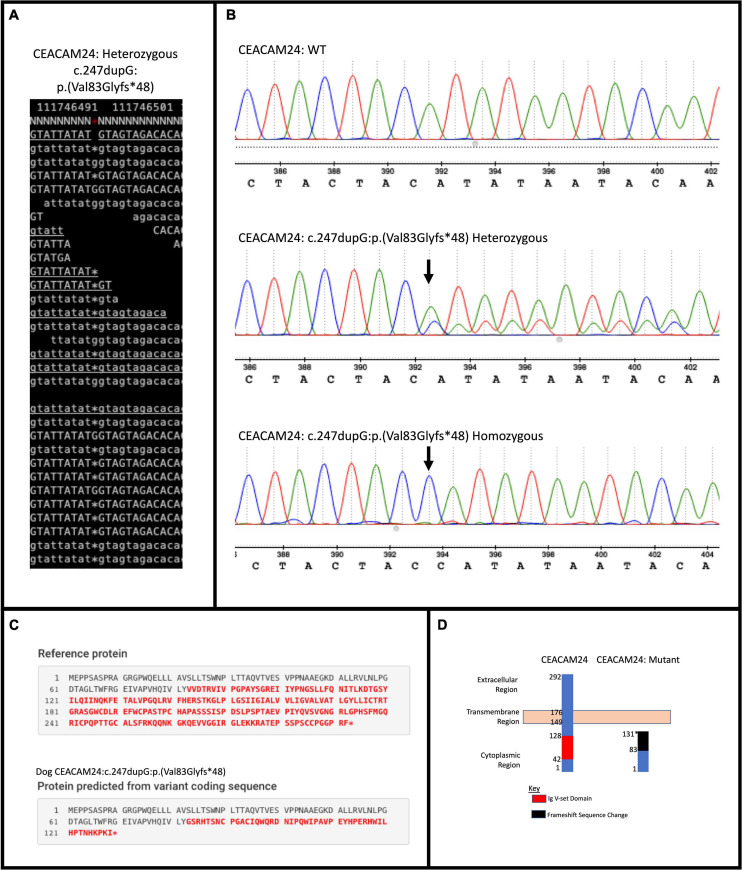
*CEACAM24* (c.247dupG; p.(Val83Glyfs*48)) mutation summary. **(A)** Samtools tview image capture of the mutation in a WGS CMT-affected Golden Retriever. **(B)** Sanger sequencing results of validation in CMT-affected Golden Retriever cohort depicting wildtype (WT), heterozygous, and homozygous sequences at the mutation location. **(C)** Mutalyzer prediction of the change in protein sequence with frame-shifting mutation. **(D)** Depiction of the WT and mutated protein and lost regions and domains of the dog CEACAM24 protein with the frame-shift mutation.

Upon filtering the WGS data, 12 different PTVs were detected in all five Golden Retrievers, four of which were within human orthologs. Only one PTV, a frame-shifting mutation in *CEACAM24* (c.247dupG;p.(Val83Glyfs^∗^48)) was determined to be a true positive upon validation (Figure 1). This mutation had a frequency of 66.7% amongst the 18 Golden Retrievers with CMT in this study (Table 1). Upon comparing that frequency to the 17.3% allele frequency in the European Variation Archive, a *p-*value of 1.52 × 10^–8^ was generated. Representing dogs from another continent and not knowing the breeds of the European Variation Archive, the frequency of *CEACAM24* c.247dupG;p.(Val83Glyfs^∗^48) was subsequently determined in different American Kennel Club breeds (Table 1). There was no statistically significant difference between Golden Retriever CMT cases and controls. However, there was a significant difference between Golden Retrievers cases and other American Kennel Club breeds (2.48 × 10^–5^; Table 1). The *CEACAM24* c.247dupG;p.(Val83Glyfs^∗^48) allele frequency ranged from 0 to 80% in the assessed breeds (Table 1). *CEACAM24* c.247dupG;p.(Val83Glyfs^∗^48) abolishes the extracellular region, the transmembrane domain, and part of the cytoplasmic region, including the Ig V-set domain (Figures 1C,D).

**TABLE 1:** *CEACAM24* c.247dupG; p.(Val83Glyfs*48) genotypes and allele frequencies.

Data set/Cohort	Dog breed	# of dogs	# of HOM	# of HET	Minor allele frequency	*p*-value for comparison to CMT affected Golden Cohort
CMT Affected	Golden Retriever	18	6	9	66.7	-
CHIC United States Breed Specific Controls	Golden Retriever	87	42	34	67.8	0.3334
CHIC United States Non-Golden Retriever Controls	Petit Basset Griffon Vendeen	10	7	2	80.0	**2.48 × 10^–5^**
	Gordon Setter	8	5	2	75.0	
	Australian Cattle Dog	10	4	2	50.0	
	Siberian Husky	10	4	1	45.0	
	Dalmatian	10	3	2	40.0	
	Irish Setter	9	0	1	5.6	
	Welsh Pembroke Corgi	10	0	0	0.0	
	Standard Schnauzer	10	0	0	0.0	
	Newfoundland	10	0	0	0.0	
	Keeshond	10	0	0	0.0	
	Great Dane	8	0	0	0.0	
	Doberman Pinscher	10	0	0	0.0	
	Boxer	10	0	0	0.0	
	Totals and Avg MAF of CHIC Non-Golden Retriever Controls	125	23	10	22.4	
European Variation Archive Controls	European General Dog Population	196	12	44	17.3	**1.52 × 10^–8^**

*The bold values represent significant *p*-values, *p*-values less than 0.05.*

**FIGURE 2:**
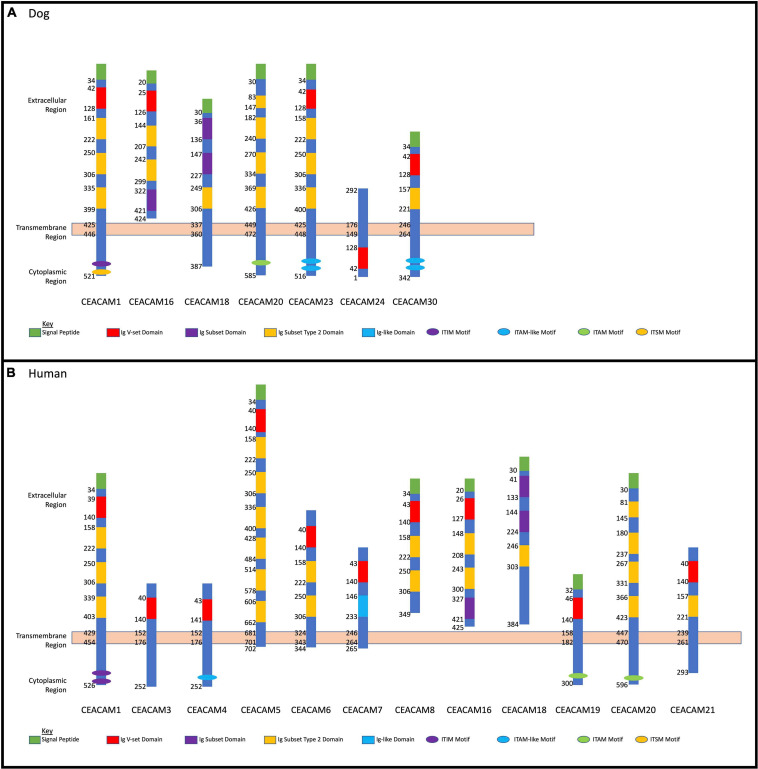
Dog and human *CEACAM* gene family protein domain analysis. **(A)** Dog CEACAM protein domain and binding site depictions with membrane regions. **(B)** Human CEACAM protein domain and binding site depictions with membrane regions.

Homology analysis revealed that the dog CEACAM proteins were, on average, 43.7% similar to the dog CEACAM24 protein (Table 2 and Figure 2A). Similarly, there were many related functional domains and high homology between the dog CEACAM24 protein and the human CEACAM proteins, averaging 51.9% similarity (Table 2 and Figure 2). This homology, along with the fact that there is no direct human ortholog of dog *CEACAM24*, prompted all human *CEACAM* genes (Figure 2B) to be investigated for rare PTVs in the TCGA breast cancer cohort.

**TABLE 2:** Homology of dog and human ceacam proteins to dog CEACAM24 protein.

Species	Gene name	Protein accession	% Identity	% Similarity
Dog	CEACAM1	NP_00101026	52.2	58.4
	CEACAM16	ENSCAFP00000039084	22.5	37.7
	CEACAM18	ENSCAFP00000058450	19.3	32.5
	CEACAM20	ENSCAFP00000036293	21.2	31.9
	CEACAM23	NP_001091021	38.4	40.8
	CEACAM24	NP_001091023	100	100
	CEACAM28	NP_001091015	42.2	46.3
	CEACAM30	NP_001091022	53.6	58.3
Average of all Dog CEACAM proteins compared to Dog CEACAM24 (excluding CEACAM24 from analysis)			35.6	43.7
Human	CEACAM1	NP_001171744	53.1	60.8
	CEACAM3	NP_001806	47	58.2
	CEACAM4	NP_001808	50.4	63.4
	CEACAM5	NP_004354	53.2	61
	CEACAM6	NP_002474	37.8	48
	CEACAM7	NP_008821	45.1	58.3
	CEACAM8	NP_001807	53.8	63.6
	CEACAM16	NP_001034302	28	43.5
	CEACAM18	NP_001265321	26.9	46.2
	CEACAM19	NP_064604	23.7	38.1
	CEACAM20	NP_001096067	25.7	39.9
	CEACAM21	NP_001091976	34.1	42.3
Average of all Human CEACAM proteins compared to Dog CEACAM24			39.9	51.9

**FIGURE 3:**
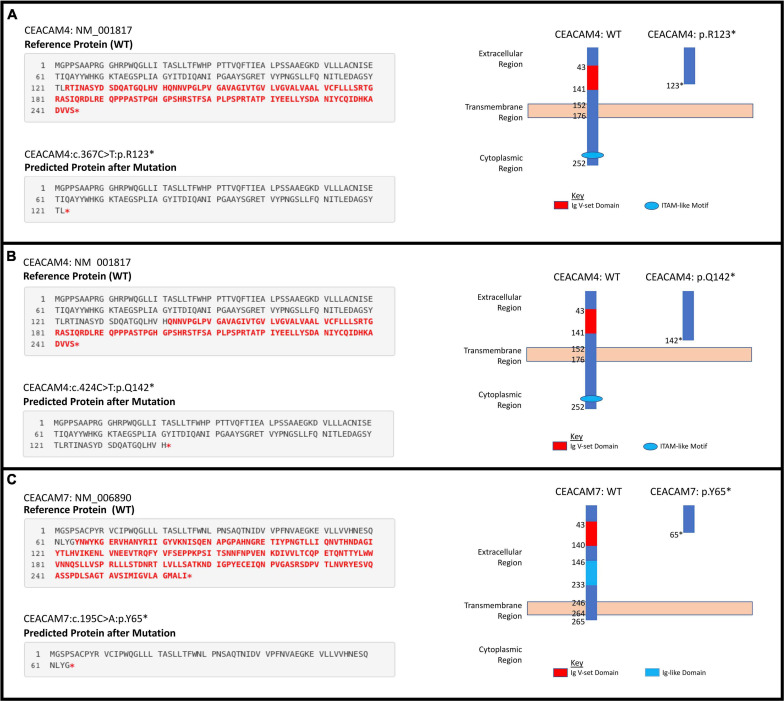
Individual significant stop gain mutations. **(A)** *CEACAM4* c.367C > T;p.(Arg123^∗^). **(B)** *CEACAM4* c.424C > T;p.(Gln142^∗^). **(C)** *CEACAM7* c.195C > A;p.(Tyr65^∗^).

**FIGURE 4:**
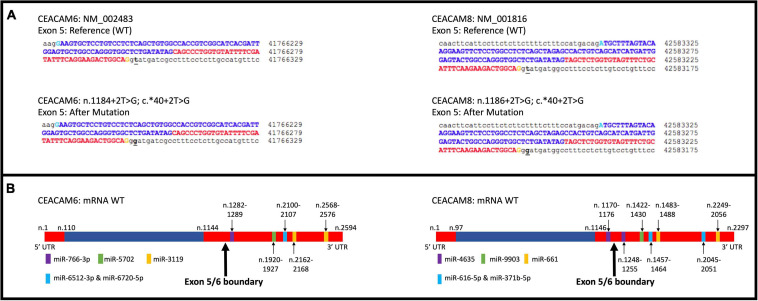
*CEACAM6* and *CEACAM8* significant splicing mutations. **(A)** Depiction of the change in genomic sequence with splice site mutation. **(B)** Depiction of the top five miRNA binding sites for *CEACAM6* and *CEACAM8* within the mature mRNA. Blue is coding and red is non-coding.

A total of six rare PTVs were identified in African Americans and sixteen in European Americans breast cancer cases (Supplementary Tables 1, 2). Single variant assessment revealed five variants associated with breast cancer risk, three of which were associated each with European and African American breast cancer (Table 3 and Figures 3, 4). One variant, *CEACAM7* c.195C > A;p.(Y65X), was associated with breast cancer risk in both ethnicities (Table 3 and Figure 3). Two stop gain mutations in *CEACAM4* were associated with African American breast cancer (Table 3 and Figure 3), and two splicing mutations were associated with European American breast cancer, one in *CEACAM6* and another within *CEACAM8* (Table 3 and Figure 4). Both of those splicing mutations affect non-protein-coding exons in the 3′ UTR, which, instead of truncating the protein, potentially disrupt key microRNA binding sites previously associated with cancer (Table 4 and Figure 4). Overall, gene-based aggregation analyses revealed that rare PTVs in *CEACAM6*, *CEACAM7*, and *CEACAM8* are associated with European American breast cancer risk, and rare PTVs in *CEACAM7* are associated with breast cancer risk in African Americans (Table 5). Ultimately, rare PTVs in the entire *CEACAM* gene family are associated with breast cancer risk in both European and African Americans with respective *p-*values of 1.75 × 10^–13^ and 1.87 × 10^–04^ (Table 5).

**TABLE 3:** Significant mutations in CEACAM gene family. Individual mutation *p*-values were calculated using Fisher’s Exact test.

Gene name	Variant type	Genomic position on Chr 19	mRNA variant name	Protein variant name	rs ID	EA			AA		
							
						MAF (%)		Mutation specific *p*-values	MAF (%)		Mutation specific *p*-values
									
						EVS EA	TCGA EA	TCGA EA	EVS AA	TCGA AA	TCGA AA
CEACAM4: NM_001817	stopgain	41625658	c.367C > T	p.R123X	rs147663846	−	−	−	0.20	0.89	**0.04803**
	stopgain	41625601	c.424C > T	p.Q142X	rs199937487	−	−	−	0.02	0.60	**0.01431**
CEACAM6: NM_002483	splicing	41766301	c.*40 + 2T > G	−	rs782698255	0.00	0.46	**7.40E-06**	0.00	0.30	0.07636
CEACAM7: NM_006890	stopgain	41687091	c.195C > A	p.Y65X	rs782316651	0.00	10.79	**2.20E-16**	0.00	4.46	**2.20E-16**
CEACAM8: NM_001816	splicing	42583204	c.*40 + 2T > G	−	rs748512513	0.00	1.62	**2.20E-16**	−	−	−

*The bold values represent significant *p*-values, *p*-values less than 0.05.*

**TABLE 4:** Top five miRNA binding sites for both *CEACAM6* and *CEACAM8* and previous cancer associations.

Gene target name	miRNA name	Previous cancer association	Previous HBOC association
CEACAM6	miR-3119	**Yes** Chen F. et al. (2018)	No
	miR-766-3p	**Yes** Chen et al. (2017); Wang Q. et al. (2017); You et al. (2018); Alshamrani (2020); Liu S. et al. (2020); Tuncer et al. (2020); Zhang et al. (2020)	**Yes** Wang Q. et al. (2017); Alshamrani (2020); Tuncer et al. (2020)
	miR-6512-3p	**Yes** Ge et al. (2020)	**Yes** Ge et al. (2020)
	miR-6720-5p	**Yes** Yasui et al. (2017); Ren et al. (2018); Ge et al. (2020)	**Yes** Ge et al. (2020)
	miR-5702	**Yes** Zhang et al. (2018); Mou and Wang (2019)	**Yes** Mou and Wang (2019)
CEACAM8	miR-661	**Yes** Vetter et al. (2010); Hoffman et al. (2014); Zhu et al. (2015); Liu et al. (2017); Wang et al. (2018); Sun et al. (2019)	**Yes** Vetter et al. (2010); Zhu et al. (2015); Wang et al. (2018); Sun et al. (2019)
	miR-9903	**Yes** Shu et al. (2018)	**Yes** Shu et al. (2018)
	miR-616-5p	**Yes** Bai et al. (2017); Wang D. X. et al. (2017); Chen Z. et al. (2018); Zhu and Li (2020)	**Yes** Chen Z. et al. (2018); Zhu and Li (2020)
	miR-371b-5p	**Yes** Li et al. (2020); Luo et al. (2020)	No
	miR-4635	**Yes** Cartier et al. (2017); Guan et al. (2017); Jiang et al. (2019); Shimojo et al. (2019); Yokoi et al. (2019)	**Yes** Guan et al. (2017)

**TABLE 5:** Aggregation analysis for rare (<1% MAF) PTVs in the *CEACAM* gene family.

Gene name	Gene specific *p*-values	
	
	AA	EA
CEACAM1: NM_001184815	1	0.8784262
CEACAM3: NM_001815	1	0.3978745
CEACAM4: NM_001817	0.148726	0.7479721
CEACAM5: NM_004363	1	0.8516203
CEACAM6: NM_002483	0.07636	**1.4423E-05**
CEACAM7: NM_006890	**1.8694E-12**	**1.2241E-11**
CEACAM8: NM_001816	0.2727805	**6.4189E-12**
CEACAM16: NM_001039213	0.923479	0.9930833
CEACAM18: NM_001278392	1	1
CEACAM19: NM_020219	1	1
CEACAM20: NM_001102597	1	0.9190567
CEACAM21: NM_001098506	0.9604724	0.7104384
***CEACAM* gene family**	**1.87E-04**	**1.75E-13**

*The bold values represent significant *p*-values, *p*-values less than 0.05.*

## Discussion

Utilizing a comparative oncology approach, our team identified *CEACAM24* c.247dupG;p.(Val83Glyfs^∗^48) in Golden Retrievers with CMT and subsequently determined that rare PTVs in the entire *CEACAM* gene family were associated with inherited breast cancer risk in humans. We previously described a large Golden Retriever pedigree with segregating CMT, carried out WGS on five selected Golden Retriever cases, and highlighted variants in orthologs of human breast cancer susceptibility genes (Huskey et al., 2020). In this current study, we used the same WGS dataset to identify novel variants that could be influencing Golden Retriever CMT susceptibility. We isolated PTVs found in all five sequenced Golden Retriever samples, and, upon validation, determined the mutation status in the 13 remaining CMT-affected Golden Retrievers within the pedigree. *CEACAM24* c.247dupG;p.(Val83Glyfs^∗^48) was the only validated variant and had an allele frequency of 66.7% amongst the 18 CMT-affected dogs. Despite not being recognized as a breed highly affected by CMT, Golden Retrievers have a higher prevalence of cancer compared to many dog breeds with 65% of Golden Retrievers in the United States succumbing to the disease (Dobson, 2013; Salas et al., 2015; Kent et al., 2018). The Golden Retriever *CEACAM24* c.247dupG;p.(Val83Glyfs^∗^48) allele frequency and cancer mortality rate are very similar.

The CMT-affected Golden Retrievers within this study can all be linked back to a sire in the United States from the 1950s, which was shortly after the registration of the breed with the American Kennel Club. Since importation to and registration in the United States, Golden Retrievers in Europe and the United States are considered two distinct populations, as breeding between the two continents is rare and unique gene pools have been established due to strict breeding standards and the popular-sire effect (Brackman, 2020). Cancer mortality in European-bred Golden Retrievers has been reported to be 38.8%, which is much lower than Golden Retrievers in the United States (65%) (Dobson, 2013; Kent et al., 2018). These differences could be explained by distinct genetic risk factors. The allele frequency of *CEACAM24* c.247dupG;p.(Val83Glyfs^∗^48) in the European Variant Archive was 17.3%, which corresponded to a *p-*value of 1.52 × 10^–8^ when compared to our CMT-affected Golden Retrievers from the United States. However, in addition to not knowing breed-specific information in the European Variant Archive, genetic bottlenecks upon importation to the United States need to be acknowledged. Thus, comparing allele frequencies to a United States dog population with known breed status was important, which can be determined through American Kennel Club registration. Overall, *CEACAM24* c.247dupG;p.(Val83Glyfs^∗^48) appears to be common in Golden Retrievers in the United States with an allele frequency of 67.8%, which is not significantly different from the CMT-affected Golden Retriever cases. However, that allele frequency was determined by screening 87 Golden Retrievers from the CHIC repository with unknown disease diagnoses and age at sample submission. This is not ideal for canine cancer studies; older dogs (> than 8 years of age) with unaffected CMT-status are recommended (Tonomura et al., 2015; Hayward et al., 2016). In saying that, if *CEACAM24* c.247dupG;p.(Val83Glyfs^∗^48) truly is a high-frequency allele in Golden Retrievers due to a genetic bottleneck in the United States, it can explain why 65% of Golden Retrievers succumb to cancer (Kent et al., 2018).

Regarding the assessment of other American Kennel Club breeds, an overall *CEACAM24* c.247dupG;p.(Val83Glyfs^∗^48) allele frequency of 22.4% was revealed, which was significantly different from CMT-affected Golden Retriever cases. Noting the small sample sizes of each breed, over half of the assessed breeds showed no presence of the variant. However, some breeds contained the variant at higher levels; most notably, Petit Basset Griffon Vendeen, Gordon Setter, Australian Cattle Dog, Siberian Husky, and Dalmatian. Petit Basset Griffon Vendeen, which had the highest allele frequency, has a cancer mortality rate of 33% (Dobson, 2013). In a United Kingdom study, Dalmatians, Gordon Setters, and Siberian Huskies were found to have cancer mortality rates ranging from 19.1 to 31.8% (Dobson, 2013), and Australian Cattle Dogs have a rate of 27% (Petmed, 2014).

*CEACAM24* is a part of the dog *CEACAM* gene family (Figure 2A), which is a subdivision of the immunoglobulin superfamily of cell adhesion molecules (IgCAMs) (Smith and Xue, 1997; Kuespert et al., 2006). All IgCAMs, and hence all CEACAM proteins, are characterized by having at least one immunoglobulin (Ig)-like domain (Figure 2). *CEACAM* genes have diverse functions in both dogs and humans, including cell-cell adhesion, cell signaling, immunity/inflammation, angiogenesis, and tumor development, progression and metastasis (Kuespert et al., 2006; Kammerer et al., 2007; Kammerer and Zimmermann, 2010; Beauchemin and Arabzadeh, 2013; Han et al., 2020). *CEACAM24* c.247dupG;p.(Val83Glyfs^∗^48) abolishes the extracellular region, the transmembrane domain, and part of the cytoplasmic region, including the Ig V-set domain; thus, it is presumed to be a loss-of-function mutation. According to Ensembl, no other stop gain or frame-shifting variants have been identified in dog *CEACAM* genes. However, one splicing mutation in *CEACAM28* (c.1415-2A > G) was identified, which had a 34% allele frequency within the European Variation Archive. The *CEACAM* gene family is present in many mammalian species but has evolved in a highly species-specific manner, heavily influenced by pathogen/host coevolution (Kammerer et al., 2007; Kammerer and Zimmermann, 2010; Weichselbaumer et al., 2011). Despite phylogenetic discordance of dog and human *CEACAM* genes (Weichselbaumer et al., 2011), our analyses revealed there is high homology between the dog CEACAM24 protein and the human CEACAM proteins, averaging 51.9% similarity. This homology, along with the fact that there is no direct human ortholog of the *CEACAM24* gene, prompted all human *CEACAM* genes to be investigated for rare PTVs in the TCGA breast cancer cohort.

There are 12 human *CEACAM* genes, all of which cluster on chromosome 19q13.2-19q13.4. Over the years, genetic markers in that region have been associated with many different types of cancer susceptibility, including breast cancer (Rockenbauer et al., 2002; Yin et al., 2002; Nexo et al., 2003, 2008; Vogel et al., 2004; Amin Al Olama et al., 2013; Gao et al., 2018). Nonetheless, inherited mutations in *CEACAM* genes have yet to be associated with inherited risk of cancer (Zheng et al., 2011; Kammerer et al., 2012; Wang et al., 2015). Aberrant expression of many *CEACAM* genes have been associated with tumorigenesis, and *CEACAM* gene products are recognized as clinically-relevant tumor markers (Kuespert et al., 2006; Beauchemin and Arabzadeh, 2013; Han et al., 2020). Regarding breast cancer, *CEACAM1* has been shown to be down-regulated compared to normal breast tissue (Yang et al., 2015), similar to its expression in prostate (Busch et al., 2002; Liu J. et al., 2020), endometrial (Bamberger et al., 1998), gastric (Takeuchi et al., 2019) and colon cancer (Fournes et al., 2001; Song et al., 2011), identifying it as a tumor suppressor. It has also been demonstrated that *CEACAM5* (Iqbal et al., 2017; Powell et al., 2018), *CEACAM6* (Maraqa et al., 2008; Tsang et al., 2013; Iqbal et al., 2017; Rizeq et al., 2018), and *CEACAM19* (Michaelidou et al., 2013; Estiar et al., 2017) are overexpressed in breast cancer and are associated with enhanced tumor invasiveness and metastasis. Conversely, *CEACAM6* and *CEACAM8* co-expression inhibits proliferation and invasiveness of breast cancer cells (Iwabuchi et al., 2019). Additionally, *CEACAM* gene splice variants have been suggested to play a role in breast cancer tumorigenesis (Gaur et al., 2008; Zisi et al., 2020). Lastly, through exome sequencing, Li et al. observed loss of heterozygosity of *CEACAM1*, *CEACAM3*, *CEACAM5*, *CEACAM6*, *CEACAM7*, and *CEACAM8* in breast cancer tumors that were associated with metastasis, suggesting that this closely-linked gene family regulates tumorigenesis and metastasis synergistically (Li et al., 2014). Corroborating those preliminary findings, we have now determined that rare inherited PTVs in the entire *CEACAM* gene family are associated with breast cancer risk in both European and African Americans with respective *p*-values of 1.75 × 10^–13^ and 1.87 × 10^–04^. The *p*-value generated for African American breast cancer risk was likely influenced by the small sample size in TCGA.

We analyzed blood-derived exomes of European and African American breast cancer cases in TCGA to identify inherited PTVs in all human *CEACAM* genes, and detected sixteen and six rare PTVs in each ethnicity, respectively. Gene-based analyses determined that rare PTVs in *CEACAM6*, *CEACAM7*, and *CEACAM8* are associated with European American breast cancer risk, and rare PTVs in *CEACAM7* are associated with breast cancer risk in African Americans. *CEACAM7*, which was associated with breast cancer risk in both ethnicities, has no current link to breast cancer. However, down-regulation of *CEACAM7* in hyperplastic polyps and early adenomas represent some of the earliest observable molecular events leading to colorectal tumors (Scholzel et al., 2000). Though CEACAM7 expression was thought to be restricted to the epithelial cells of the colon and pancreas, according to the Human Protein Atlas, grandular cells of the breast have moderate CEACAM7 protein expression (Uhlen et al., 2015; Raj et al., 2021). How *CEACAM7* plays a role in breast cancer is currently unknown, but the link could even be indirect and due to expression in non-breast tissue (Ferreira et al., 2019). *CEACAM7* c.195C > A;p.(Y65X), which was detected in 10.8 and 4.5% of European and African American cases, respectively, was absent in all EVS controls. It severely truncates the 265 amino acid proteins and results in a loss of the cytoplasmic region, as well as a large portion of the extracellular region, including disruption of the Ig-like and Ig V-set domains. It is likely a loss-of-function mutation (Figure 3).

Rare PTVs in *CEACAM6* and *CEACAM8* appear to only be associated with European American breast cancer risk. Considering that *CEACAM6/8* co-expression inhibits proliferation and invasiveness of breast cancer cells (Iwabuchi et al., 2019), having a rare PTV in one of those two genes may be sufficient to override their synergistic tumor-suppressing relationship. While a number of PTVs were detected in these genes, two splicing mutations, *CEACAM6* c.^∗^40 + 2T > G and *CEACAM8* c.^∗^40 + 2T > G, were individually determined to be associated with European American breast cancer, both of which affect non-coding exons in the 3′ UTR. Both mutations affect the donor site immediately following exon 5 of their respective genes, which contains both coding and non-coding DNA. The mutated donor sites likely affect the downstream sequence of the mature mRNA product, either retaining (all or a part of) intron 5 or removing exon 6, the last non-coding exon, where many microRNA binding sites are located (Figure 4). Based on miRDB rankings, the top five microRNAs that bind to the 3′ UTRs of *CEACAM6* and *CEACAM8* have previous links to cancer (Table 4); thus, disrupted microRNA binding likely leads to aberrant *CEACAM6* and *CEACAM8* expression.

Two stop gain mutations in *CEACAM4* (c.367C > T;p.R123X and c.424C > T;p.Q142X) were associated with African American breast cancer. These mutations were not detected in European American cases or controls, and are very rare in the general African American population. They were detected in significantly more African American breast cancer cases compared to ethnic-matched controls, suggesting their involvement in African American breast cancer risk. However, gene-based aggregation analyses did not support *CEACAM4* as a breast cancer risk gene. Larger African American breast cancer cohorts will need to be studied to validate these findings. Interestingly, in a study of parous women with and without breast cancer, *CEACAM4* has been reported to be up-regulated in normal breast compared to breast tumor samples (Balogh et al., 2007). Though race/ethnicity was not revealed in that study, the results suggest that *CEACAM4* could be a breast cancer tumor suppressor.

It has long been reported that minimal genetic changes can have radical effects on the function of *CEACAM* genes (Naghibalhossaini and Stanners, 2004). Residues in CEACAM6 and CEACAM8 have been identified that are critical for CEACAM6 homodimerization as well as the formation of *CEACAM6* and *CEACAM8* heterodimers, which is important in preventing breast cancer cell proliferation (Kuroki et al., 2001; Iwabuchi et al., 2019). There have also been residues reported in *CEACAM1* that are crucial for determining the risk of infection by receptor-binding pathogens (Villullas et al., 2007) and preventing the killing activity of NK cells (Markel et al., 2004). Furthermore, somatic missense mutations in colorectal cancers have been detected in *CEACAM1* (Song et al., 2011) and *CEACAM5* (Gu et al., 2020), the latter of which has been shown to increase proliferation by inhibiting TGFB signaling and altering the intestinal microbiome. The microbiome has been reported as a new breast cancer risk factor (Fernandez et al., 2018; Eslami et al., 2020). In fact, differences have been reported in the microbiome of normal and cancerous breast tissue, as well as the gut microbiota of breast cancer cases versus controls (Fernandez et al., 2018). Disrupted *CEACAM* genes could be the underlying mechanism through altered TGFB signaling, bacteria docking, and/or estrogen metabolism (Villullas et al., 2007; Tchoupa et al., 2014; Fernandez et al., 2018; Gu et al., 2020). This study reports the first association of inherited *CEACAM* mutations and breast cancer risk, and potentially implicates the whole gene family in genetic risk. Precisely how these mutations contribute to breast cancer needs to be determined, especially considering our current knowledge on the role that the *CEACAM* gene family plays in tumor development, progression, and metastasis.

## Data Availability Statement

The WGS data for the five whole genome sequenced CMT-affected Golden Retriever dogs can be obtained through the NCBI SRA repository through BioProject PRJNA745215. TCGA data is available through dbGAP.

## Ethics Statement

The studies involving human participants were reviewed and approved by Auburn University Institutional Review Board (IRB) for the Protection of Human Subjects in Research. The patients/participants provided their written informed consent to participate in this study. Ethical review and approval was not required for the animal study because this research did not require ORC – Animal Care & Use (IACUC) approval since only dog DNA was studied upon receipt from the CHIC repository.

## Author Contributions

AH and NM wrote the manuscript and performed variant and statistical analyses. AH and IM performed PCR for validation and determining mutational frequency. AH performed bioinformatic processing. All authors read and approved the final manuscript.

## Conflict of Interest

The authors declare that the research was conducted in the absence of any commercial or financial relationships that could be construed as a potential conflict of interest.

## Publisher’s Note

All claims expressed in this article are solely those of the authors and do not necessarily represent those of their affiliated organizations, or those of the publisher, the editors and the reviewers. Any product that may be evaluated in this article, or claim that may be made by its manufacturer, is not guaranteed or endorsed by the publisher.

## Footnotes

1
https://www.ebi.ac.uk/eva/?eva-study=PRJEB24066

## Abbreviations

CMT: canine mammary tumor
PTV: protein truncating variant
CHIC: Canine Health Information Center
WGS: whole genome sequencing
PCR: Polymerase chain reaction
ELM: Eukaryotic Linear Motif
TCGA: The Cancer Genome Atlas
SNVs: single nucleotide variants
BAM: binary sequence alignment mapping
GDC: Genomic Data Commons
GATK’s: Genome Analysis Toolkit’s
gVCF: genome variant calling format
NHLBI: NationalNational Heart, Lung, and Blood Institute
HBOC: hereditary breast and ovarian cancer.
